# Dewetting Metal Nanofilms—Effect of Substrate on Refractive Index Sensitivity of Nanoplasmonic Gold

**DOI:** 10.3390/nano9111530

**Published:** 2019-10-27

**Authors:** Nikhil Bhalla, Aditya Jain, Yoonjoo Lee, Amy Q. Shen, Doojin Lee

**Affiliations:** 1 Nanotechnology and Integrated Bioengineering Centre (NIBEC), School of Engineering, Ulster University, Shore Road, Jordanstown BT37 0QB, Northern Ireland, UK; n.bhalla@ulster.ac.uk; 2 Seagate Technology LLC, 7801 Computer Ave, Bloomington, MN 55435, USA; aditya.jain@seagate.com; 3 Energy Efficiency Materials Center, Korea Institute of Ceramic Engineering and Technology, 101 Soho-Ro, Jinju-Si, Gyeongsangnam-Do 52851, Korea; yoonjoo_lee@kicet.re.kr; 4 Micro/Bio/Nanofluidics Unit, Okinawa Institute of Science and Technology Graduate University, Onna, Okinawa 904-0495, Japan; 5 Department of Polymer Science and Engineering, Chonnam National University, Gwangju 61186, Korea; 6 Fibrous Ceramics and Aerospace Materials Center, Korea Institute of Ceramic Engineering and Technology, 101 Soho-Ro, Jinju-Si, Gyeongsangnam-Do 52851, Korea

**Keywords:** dewetting, metal nanofilm, localized surface plasmonic resonance, refractive index sensitivity

## Abstract

The localized surface plasmon resonance (LSPR) sensitivity of metal nanostructures is strongly dependent on the interaction between the supporting substrate and the metal nanostructure, which may cause a change in the local refractive index of the metal nanostructure. Among various techniques used for the development of LSPR chip preparation, solid-state dewetting of nanofilms offers fast and cost effective methods to fabricate large areas of nanostructures on a given substrate. Most of the previous studies have focused on the effect of the size, shape, and inter-particle distance of the metal nanostructures on the LSPR sensitivity. In this work, we reveal that the silicon-based supporting substrate influences the LSPR associated refractive index sensitivity of gold (Au) nanostructures designed for sensing applications. Specifically, we develop Au nanostructures on four different silicon-based ceramic substrates (Si, SiO_2_, Si_3_N_4_, SiC) by thermal dewetting process and demonstrate that the dielectric properties of these ceramic substrates play a key role in the LSPR-based refractive index (RI) sensitivity of the Au nanostructures. Among these Si-supported Au plasmonic refractive index (RI) sensors, the Au nanostructures on the SiC substrates display the highest average RI sensitivity of 247.80 nm/RIU, for hemispherical Au nanostructures of similar shapes and sizes. Apart from the significance of this work towards RI sensing applications, our results can be advantageous for a wide range of applications where sensitive plasmonic substrates need to be incorporated in silicon based optoelectronic devices.

## 1. Introduction

In recent years, nanoplasmonic materials have received much attention on account of their exclusive properties owing to their strong interactions with electromagnetic radiations and subsequent resonant excitations of conduction electrons [1,2,3]. Nanoplasmonic materials consist of nanostructures where the free electron oscillation of the metal can be coupled with electromagnetic radiation with much larger wavelength, compared to the size of the nanostructures [4]. These materials provide good foundation for enabling technologies associated with biosensing [5,6], plasmonic photocatalysis [7,8], solar cells and energy harvesting [9,10], optical antennas [11,12] and imaging systems [13,14,15]. The most important characteristic feature associated with nanoplasmonic structures is the localized surface plasmon resonance (LSPR). LSPR results from the collective oscillation of the conduction electrons owing to interactions between the incident light and the conduction band electrons of the metal. It leads to a strong electric field enhancement around the nanostructures by squeezing electromagnetic energy into subwavelength spatial regions [3,4,16]. It is well known that the LSPR sensitivity of the metal nanostructures on a dielectric substrate is strongly dependent on the size, spacing and the shape of these nanostructures. Moreover, the morphological and electronic properties of the supporting substrate on which plasmonic nanostructures are fabricated also affects the LSPR in nanostructures. However, how plasmonic nanostructures interact with their supporting substrates is not well established in literature [16,17,18]. Theoretically, the interaction between the supporting substrate and the metal nanostructures may cause a change in the refractive index environment of the nanostructure, thereby delocalizing the field into the substrate. This is because the electric field of the localized plasmon resonance is highly dependent on the dielectric property of the surrounding environment, and thus the supporting substrate with different dielectric characteristics may affect the plasmonic properties of the metal nanostructures [16]. For instance, Michelle et al. showed that chemical interactions at the interface between the nanoparticle and the substrate contribute significantly to the differences observed in the experimental and theoretical LSPR sensitivities [18]. Very recently, Bhalla et al. revealed that minute differences in the thickness and composition of silicon nitride led to large variations in LSPR-exciton coupling between gold (Au) nanostructures and insulator semiconductor nanojunctions [19]. Similarly, Knight et al. showed spectral shifts and symmetry breaking of the LSPR by two adjacent dielectric substrates, inducing a plasmon hybridization [20,21]. In contrast, no plasmon hybridization occurred when metal nanostructures were embedded in a uniform dielectric medium.

Among various nanoplasmonic fabrication techniques, such as nanosphere lithography [18], e-beam lithography [22], plasma synthesis [23], focused ion beam machining [24], chemical deposition [25] and solid-sate dewetting [26], the dewetting procedure offers cost effective and time efficient solutions for fabrication of the plasmonic nanostructures on a given substrate. However, it should be noted that these advantages of dewetting are also associated with the trade off with relatively less periodically distributed nanostructures. Nevertheless the substrates still demonstrate good sensitivity for bio/chemical sensing applications. Essentially, dewetting protocols involve deposition of a metal thin film, followed by annealing at temperatures below melting point of the metal. Upon annealing, the metal film breaks up into hemispherical nanoislands (NI) on the substrate, where the morphological features of the NI dependent on the interaction between the substrate and the metal film. Therefore, it is expected that dewetting of a given metal film with identical conditions on different substrates can yield nanostructures with different shapes, sizes and spacing, implying that the proper choice of the substrate for the fabrication of LSPR nanostructures is crucial for achieving LSPR chips with high sensitivity.

To reveal the aforementioned substrate effects on the LSPR chips, we have developed Au nanoplasmonic structures on four different silicon-based (Si, SiO_2_, Si_3_N_4_, SiC) ceramic substrates in large surface areas, with high throughput production. We demonstrate that the plasmonic shifts and the emergence of new plasmon modes are dependent on the spatial electron redistribution owing to the interaction between the gold nanoislands (AuNI) and the silicon-based ceramic substrates. For AuNI on Si, SiO_2_ and Si_3_N_4_, plasmonic shifts of less than 50 nm are observed on the absorbance spectra. In contrast, AuNI on the SiC substrate exhibit significantly higher plasmonic shifts of 247.80 nm/RIU (refractive index units), accompanied by the emergence of multi-mode plasmonic resonance peaks. We further perform numerical simulations to reveal the LSPR effect on different silicon-based ceramic substrates. Finally, the relationship between the surface energy and dewetting on the substrates is analyzed to comprehend the growth and formation of the AuNI, the morphology of which affects the LSPR response. Our results are of great importance for understanding the role of supporting substrates for plasmonic nanomaterials in refractive index (RI) sensing performance.

## 2. Materials and Methods

### 2.1. Reagents

All chemicals were of analytical grade and used as purchased, unless specified. Double deionised water, 18.2 MΩ cm, filtered with a Pyrogard filter (Millipore, San Antonio, TX, USA) was used to prepare all aqueous solutions. Silicon based polymers and oils for polydimethylsiloxane (PDMS) were purchased from Dow Corning, Japan. Silicon <100> wafers used for the growth of oxide, nitride, carbide and later Au nanostructures were of p-type doped with boron and purchased from Prime Wafers, Netherlands. Organic solvents such as acetone, isopropanol, methanol and glycerol solutions were all purchased from Nacalai Tesque Inc., Japan.

### 2.2. Nanoplasmonic Substrate Preparation

**Figure 1:**
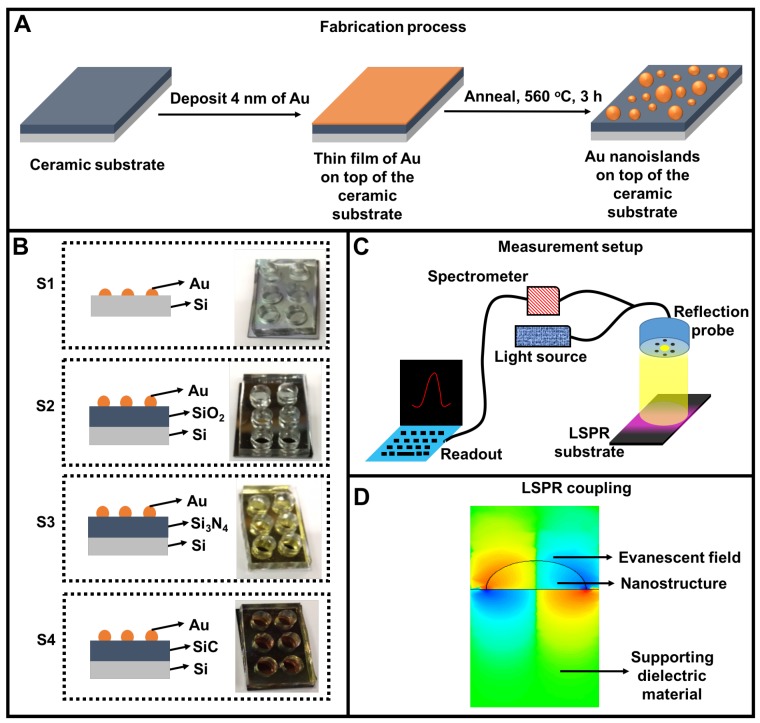
Fabrication and integration of the localized surface plasmon resonance (LSPR) chip and its measurement setup. (**A**) Two step nanoisland fabrication process where a thin film of Au is deposited on the ceramic substrate, followed by the dewetting of the Au film at high temperature. (**B**) Four types of LSPR chips where Au nanoislands are fabricated on four silicon-based ceramic substrates: silicon (S1), silicon dioxide (S2), silicon nitride (S3) and silicon carbide (SiC); (**C**) The LSPR measurement setup illustrates that LSPR wavelength shifts are acquired by the reflection of light from the top surface of the respective ceramic substrate; (**D**) Coupling of evanescent field in the supporting substrate. The strength of the coupling depends on the dielectric properties of the supporting substrate.

Silicon wafers were first rinsed with acetone and isopropanol. Next, the polished surface of the silicon wafer was deposited with 100 nm of oxide, nitride and carbide using physical chemical vapor deposition technique to create 3 different types of insulator semiconductor (IS) structures. A 4 nm thin layer of Au was deposited on the developed IS structures. The thickness of Au film was confirmed with atomic force microscopy (AFM) after establishing the tooling factors of the evaporator as also described in literature reports [27,28]. The Au thin films were then annealed at 560 °C for 3 h to produce AuNI on these supporting substrates [4,29]. The fabricated AuNI nanoplasmonic substrates were integrated with Polydimethylsiloxane (PDMS) wells. In order to fabricate PDMS wells, we first made slabs of PDMS by using 10:1 polydimethylsiloxane (PDMS) and then cured it for 3.5 h at 60 °C after a degassing process to remove air bubbles. We then punched holes of 6 mm in diameter by using disposable biopsy punchers (Kai Medical, Tokyo, Japan) to create PDMS wells. Both the PDMS and the AuNI-ceramic substrates were then exposed to oxygen plasma (Harrick Plasma, Ithaca, NY, USA) at 30 W for 1 min. After plasma treatment, PDMS well and AuNI-ceramic substrates were immediately brought in close contact to ensure strong bonding. Thereafter these LSPR chips were ready for refractive index sensing. The basic architecture and schematic of the experimental methods and observations are illustrated in Figure 1. Essentially, Figure 1A shows the schematic of the fabrication process of NI with 2 key steps—the deposition of 4 nm of Au on the supporting ceramic substrate, subsequent annealing step at 560 °C for 3 h. Figure 1B shows the cross-section and multi-welled LSPR chip developed on 4 different Si-based ceramic substrates. Figure 1C displays a simple LSPR measurement setup with sensor response in reflection mode. The LSPR wavelength shifts are obtained by the reflection of light from the top surface of the respective ceramic substrate. The evanescent fields on the AuNI associated with LSPR is shown in Figure 1D. The strength of the coupling depends on the dielectric properties of the supporting substrate. Electric fields penetrate the supporting dielectric material which influence the overall resonance of the plasmons present in the AuNI.

### 2.3. Modeling

Simulations for the plasmonic particle array were carried out using electrostatic simulation software, finite element solver, from computer simulation technology (CST). Au was modeled as a drude metal with data taken from the Handbook for optical Constants of Solids [30]. Complex permittivity for Si, SiO_2_ SiC, Si_3_N_4_ is considered to be dispersive within the frequency range of interest and taken from the weblink [31] and references within. To simulate an infinite array, we use a periodic boundary condition along the X and Y directions in simulations. Each unit cell is considered smaller than the lowest diffraction order and therefore only the specular component propagates in the free space as a plane wave. The transmitted and reflected plane wave is absorbed using an impedance matched port and the total reflected and transmitted power is normalized by the total input power.

### 2.4. Refractive Index Sensing

The instrument for LSPR characterization consists of a reflection probe (R400-7UV-VIS), a halogen light source (LS-1-LL), and a spectroscope (USB4000-UV-VIS-ES) purchased from the Ocean Optics. Before we obtain any signal from the spectroscope, the instrument was calibrated in dark and light spectrum modes. The LSPR signal was then recorded in absorption mode to obtain the wavelength dependence of the light absorbed by AuNI via the Spectrasuite software (cross-platform spectroscopy operating software from Ocean Optics). The refractive index was measured by using glycerol solutions of varied concentrations between 20–100 wt%.

### 2.5. Electron Microscopy

The scanning electron microscopy (SEM) was carried out by using a field emission scanning electron microscope (FEI Quanta 25 FEG, Tokyo, Japan). Images were acquired with a magnification of 100,000× at 10 kV, inside a vacuum chamber maintained at a pressure of 10^−4^ Pa. To avoid charging on the SiO_2_ surfaces during imaging, the glass slides were coated with a few angstroms of atomic layers of Pt/Pd.

### 2.6. Data Analysis

The diameter, spacing and aspect ratio (AR) of the AuNI were extracted from the raw SEM images by using built-in functions of image analysis software ImageJ. GraphPad Prism 7 and Orgin Pro 2015 were used for plotting graphs in the figures.

## 3. Results and Discussion

### 3.1. Morphological Characterizations

**Figure 2:**
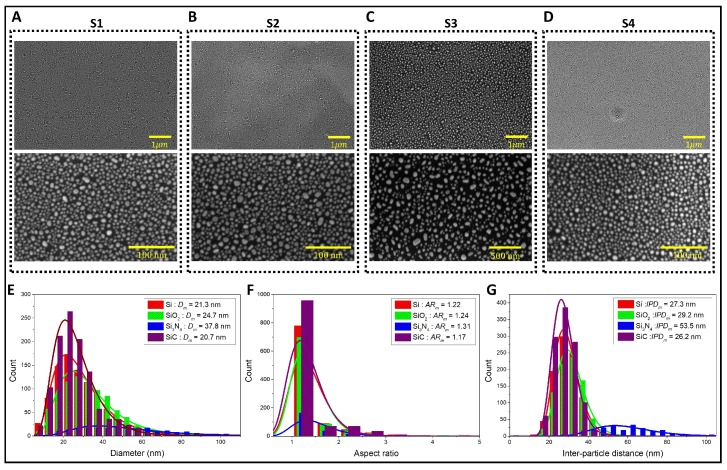
Gold nanostructure deposition on 4 different Si-based ceramic LSPR chips. (**A**) S1 = Si, (**B**) S2 = SiO_2_, (**C**) S3 = Si_3_N_4_ and (**D**) S4 = SiC substrates. Histograms and lognormal distribution functions of (**E**) particle diameter (*D*), (**F**) aspect ratio (AR) and (**G**) inter-particle distance (IPD) on 4 different supporting substrates. *D* represents the mode values of negatively skewed distributions.

Under the same fabrication protocol, the particle size (*D*), aspect ratio (AR) and inter-particle distance (IPD) of AuNI on different Si-based ceramic substrates varied owing to the different dewetting properties between Au and the supporting substrate material (Figure 2). Nevertheless, Au nanostructures were evenly deposited on four different silicon based ceramic substrates (upper panels in Figure 2A–D). These nanostructures were found to be as small as 20 nm on Si, SiO_2_ and SiC substrates, while much larger in average sizes in the case of Si_3_N_4_ (Figure 2E). Similarly, the average aspect ratio of AuNI on the Si_3_N_4_ was higher than those on the other substrates (Figure 2F). The most important parameters for enhancing the LSPR efficiency are the particle size and inter-particle distance of plasmonic AuNI. Here, the AuNI on the SiC substrate have the smallest size and the shortest inter-particle distance (Figure 2E,G), which may trigger enhanced nanoplasmonic behavior on the SiC substrate in comparison with other Si-based supporting substrates.

The growth process of nanostructures and their influence on the size distribution have been widely studied [32,33]. In general, the particle size distribution for aerosols, emulsions and powder systems are well described by a lognormal distribution,

(1) $$f\left(r\right)=\frac{A}{\sqrt{2\pi }\ln\sigma }\exp\left\{-\frac{{\left(\ln r-\ln\mu \right)}^{2}}{2{\left(\ln\sigma \right)}^{2}}\right\},$$

where μ is the location parameter, σ is the scale parameter and *A* is the concentration factor, respectively. A lognormal distribution can be applied to particle growth process where the basic mechanism is molecular diffusion that drifts through a finite growth region [33,34,35]. The distribution of AuNI (the particle sizes, aspect ratios and inter-particle distances) in our experiments can be described by the lognormal distribution, with two geometric parameters, μ and σ, being listed in Table 1. Similarly, it was reported that initial nucleation from a vapor phase to a condensed phase by absorption and coagulation would generate a low mean and narrow particle size distributions [36].

### 3.2. Surface Energy and Dewetting

An initially planar film begins to create grooves where the grain boundaries meet the surface exposed in the air and the supporting ceramic substrate, thereby, hole formation and growth lead to separated islands via surface-diffusion-controlled transport phenomenon [37]. The free energy (ΔG) of the system determines the creation of an island with radius *r*. Four surface energy terms include the surface energy per area on the substrate ($\gamma_{s}\pi r^{2}$), the surface energy per area of the island-substrate interface ($-\gamma_{i}\pi r^{2}$), the surface energy per area on the island ($\gamma_{f}\pi r^{2}$) and the isotropic surface energy associated with the edge of the island ($\gamma_{f}2\pi rh$), compete with each other to determine the total free energy of the system. Here, $\gamma_{s}$, $\gamma_{i}$ and $\gamma_{f}$ are the surface tensions of the substrate, the island-substrate interface and the island, respectively. The growth and formation of these initial holes are determined by the free energy of the system ∂ΔG/∂r where ∂ΔG/∂r decreases as the size of the hole (*r*) increases, provided that [26,37]

(2) $$r>\frac{h\gamma_{f}}{\gamma_{f}-\gamma_{i}-\gamma_{s}}.$$

If $\gamma_{s}<\gamma_{i}+\gamma_{f}$, the film dewets upon the condition that the rates of the necessary kinetic processes are sufficiently high. When $\gamma_{s}>\gamma_{i}+\gamma_{f}$, the film is stable and does not dewet. Although the contact angle (CA) on the substrate does not represent qualitative values of the surface energy of the solid substrate, we can infer that lower contact angle substrate possesses higher surface energy. Based on the CA measurements, the surface energies of the supporting substrates are found to be $\gamma_{s}^{Si_{3}N_{4}}>\gamma_{s}^{Si}∼\gamma_{s}^{SiO_{2}}>\gamma_{s}^{SiC}$. Therefore, the surface dewettability is the highest on the SiC substrate and the smallest on the Si_3_N_4_ substrate. According to Equation (2), the island radius *r* of Si_3_N_4_ would be larger than that of SiC and would be smaller than other supporting substrates. The prediction is in good agreement with the experimental results (Figure 2).

**Figure 3:**
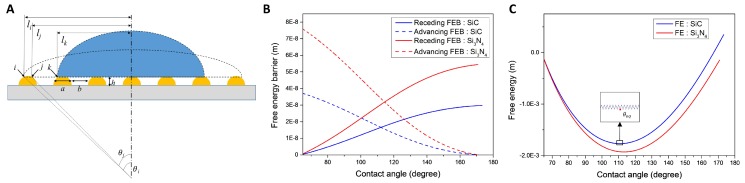
A thermodynamic approach for estimating the free energy and free energy barrier on SiC and Si_3_N_4_ substrates. (**A**) A schematic illustration of the cross-sectional configuration of a liquid drop on AuNI covered substrate; (**B**) Receding and advancing free energy barriers; and (**C**) Free energy of a liquid drop on the Au nanostructure deposited SiC and Si_3_N_4_ substrates. Inset graphs show the variation in the local energy barriers, indicating metastable and stable regions.

In many cases, nano- or micro-scale surface roughness with regular and uniform patterns can improve the hydrophobicity of a surface due to air pockets under the liquid droplet. Thermodynamic analysis is useful to investigate the surface roughness effect on the improved hydrophobicity [38,39,40,41,42]. For the AuNI patterned system, the corresponding geometrical parameters and free energy differences upon the composite Cassie state are expressed as

(3) $$\theta_{i}\frac{l_{i}^{2}}{\sin^{2}\theta_{i}}-l_{i}^{2}\cot\theta_{i}=\theta_{j}\frac{l_{j}^{2}}{\sin^{2}\theta_{j}}-l_{j}^{2}\cot\theta_{j},$$

(4) $$F_{i\to j}/\gamma^{f}=\theta_{j}\frac{l_{j}}{\sin\theta_{j}}-\theta_{i}\frac{l_{i}}{\sin\theta_{i}}+a\cos\theta_{Y},$$

(5) $$\theta_{j}\frac{l_{j}^{2}}{\sin^{2}\theta_{j}}-l_{j}^{2}\cot\theta_{j}=\theta_{k}\frac{l_{k}^{2}}{\sin^{2}\theta_{k}}-l_{k}^{2}\cot\theta_{k},$$

(6) $$F_{j\to k}/\gamma^{f}=\theta_{j}\frac{l_{j}}{\sin\theta_{j}}-\theta_{k}\frac{l_{k}}{\sin\theta_{k}}+b,$$

where, *a*, *b* and *h* are the width, inter-particle distance and height of the AuNI based on experimental measurements, respectively (Figure 3A). The imposed initial conditions for the static contact angle on a flat Au surface and the diameter of a water drop are 62.6° and 0.01 m, respectively [43]. Equations (3) and (5) are directly derived by the geometrical parameters of the nanostructures. When the edge of the meniscus of a drop moves from position *i* to *j* and *j* to *k*, the free energy differences are given by Equations (4) and (6). Next, Equations (4) and (6) are used to calculate the free energy barriers (FEBs) and the equilibrated contact angles depending on the geometric parameters of AuNI. The thermodynamic approach is used to find the stable and metastable states with the minima of the free energy for the corresponding geometrical configurations (Figure 3B). The supporting substrate Si_3_N_4_ shows higher advancing and receding FEBs than those of SiC, thus producing more stabilized hydrophobic conditions for the Si_3_N_4_ substrate. The equilibrated contact angle can be determined by intersecting the advancing and receding curves (Figure 3C). The free energy has its minima at 113.1° and 110.8° for the AuNI covered Si_3_N_4_ and SiC substrates, respectively. These predictions are in good agreement with the experimental results (see Table 2 and Supplementary Information Figure S1).

Overall, the AuNI structure on the Si-based ceramic substrates increases the contact angle due to the Cassie state in which air pockets between the nanostructures impede wetting on the substrates. These results imply that the ceramic substrates with AuNI should enhance LSPR sensitivity by improved dewettability of the Au film on the substrates owing to uniform and narrow AuNI size distributions. Nonetheless, the plasmonic efficiency may differ depending on the local dielectric properties of the AuNI on different ceramic substrates, which will be discussed in Section 3.4.

### 3.3. LSPR Characterization

**Figure 4:**
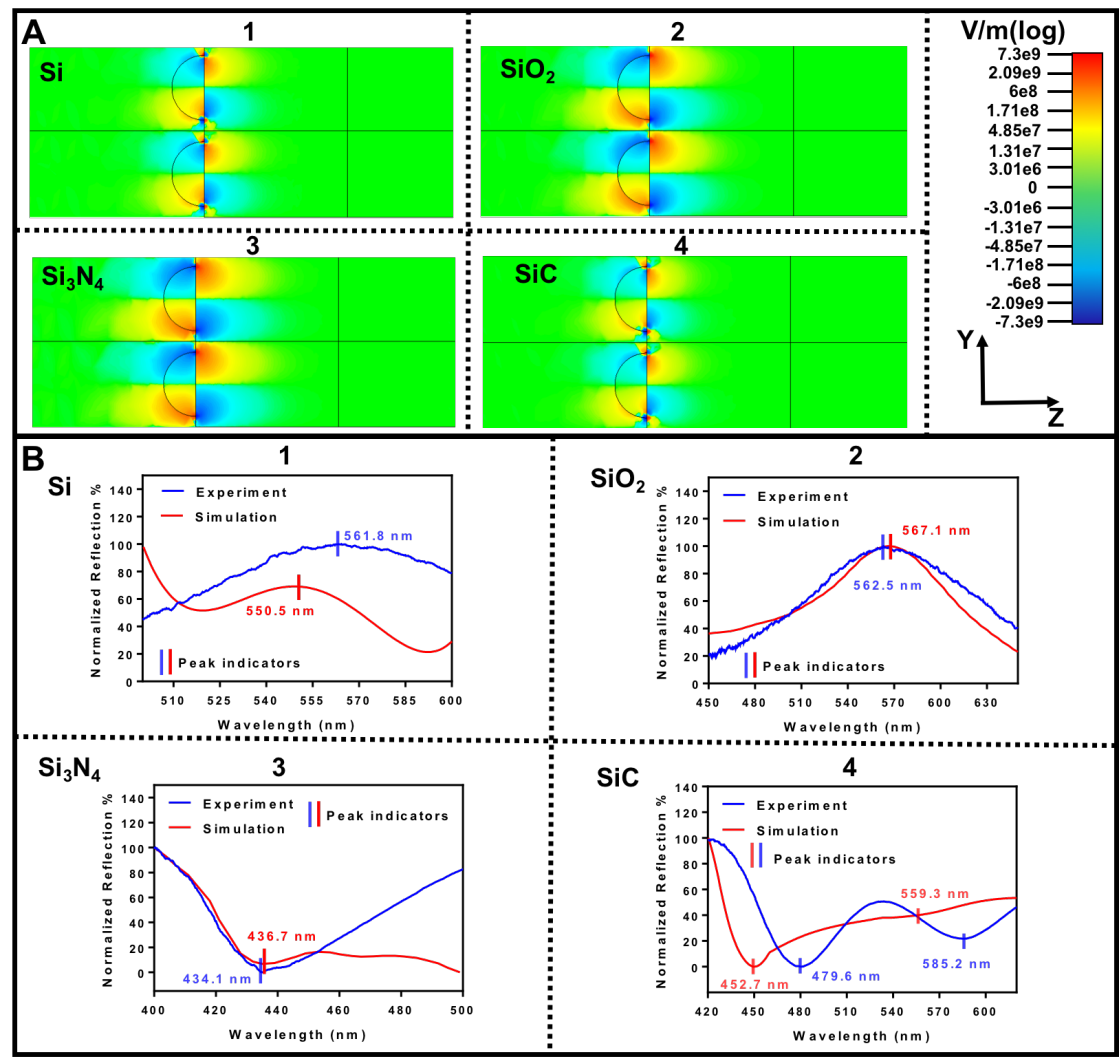
Finite element simulations: (**A**) shows the electric field of plasmons on AuNI and its penetration inside the supporting substrates (1) Si, (2) SiO_2_, (3) Si_3_N_4_ and (4) SiC; (**B**) exhibits the wavelength of the electron oscillations compared with the experimental measurements for LSPR in (1) Si, (2) SiO_2_, (3) Si_3_N_4_ and (4) SiC.

Figure 4A shows the simulations for the plasmonic nanoisland array carried out using the electromagnetic (EM) simulation software from CST technologies. As seen from the SEM characterization, the AuNI are randomly dispersed on the supporting substrates and the radius distribution of AuNI also varies. To emulate the experimental conditions, we take the radius of the nanostructures as the mean value from the experiments, extracted from a Gaussian mean of the size distribution. Inter-particle distance is also extracted from the mean. The plasmonic particle is modelled as a hemisphere, on top of a thin dielectric film, which in turn sits on a semi-infinite Silicon substrate. Fields displayed in Figure 4 are taken at the vertical marker ’peak indicator’ in each figure. The markers coincide with the resonance which is estimated from the absorbance spectra in the simulations A = 1 − T − R, where A is the absorbance, T is the transmission and R is the reflection. Figure 4A shows the Y component of the electric field simulation to depict the plasmon field inside and around the plasmonic AuNI. To understand the resonance conditions of these plasmonic AuNI, we first describe the extinction spectra for homogeneous AuNI spheres. The derivation is carried out by assuming that the AuNI is spherical and much smaller than the wavelength of the incident light. In this limit, we can approximate the scattering cross-section by a first order multipole expansion of the Mie theory as:

(7) $$\sigma_{s}=\frac{3}{2\pi }\left(\frac{\omega^{4}}{c}\right){\left(\epsilon_{d}V\right)}^{2}\left(\frac{{\left(\epsilon_{m}^{\prime }-\epsilon_{d}\right)}^{2}+{\left(\epsilon_{m}^{\prime \prime }\right)}^{2}}{{\left(\epsilon_{m}^{{}^{\prime }}+2\epsilon_{d}\right)}^{2}+{\left(\epsilon_{m}^{\prime \prime }\right)}^{2}}\right).$$

Here ω is the angular frequency of the incoming light, *c* is the speed of light, *V* is the volume of the metal sphere. $\epsilon_{m}^{\prime }$ is the real part of the permittivity of metal at that incident frequency, $\epsilon_{m}^{\prime \prime }$ is the imaginary part of the permittivity of metal at that incident frequency and $\epsilon_{d}$ is the real part of the permittivity of the surrounding dielectric medium. We assume that the AuNI is uniformly surrounded by the same dielectric material. Note that our experimental conditions differ slightly from the idealized model given in Equation (7). For instance, our AuNI is hemispherical and is surrounded by air on top and the high index dielectric substrate on the bottom, thereby experiencing an inhomogeneous photonic environment. Nevertheless, with our assumptions, we can still obtain the general trends in our spectra from Equation (7), which shows that the extinction spectra depends strongly on the surrounding dielectric substrate. The resonance occurs when $\epsilon_{m}^{\prime }$ = $-2\epsilon_{d}$. Thus, resonance shift will occur if we change the permittivity of the thin film, shown by the decay of the evanescent field in Figure 4A (1,2,3,4). It should be noted that fields in the Figure 4A coincide with the resonance of the nanostructures at wavelength shown in Figure 4B. For the SiC substrate, the field plot in Figure 4A shows the resonance at 452.7 nm. The second resonance at 559.3 nm is relatively weak in simulations, which is attributed to guided modes in the SiC substrate as also observed for silver nanostructures in the literature [44,45]. For low index dielectrics such as SiO_2_, the electric field decays slowly, while for high index dielectrics such as SiC, the electric field decay is much faster. Similarly, we can explain the extinction spectra in Figure 4B (1,2,3,4) by Equation (7). It should be noted that the discrepancy between the simulated and the experimental values of peaks Si (2.05%), SiO_2_ (0.81%), SiC (maximum of 5.94%) and Si_3_N_4_ (0.59%) is attributed to the impurities present in different dielectric substrates (not considered in simulations) in the form of fixed charges, mobile and trapped charges. A detailed characterization of the substrate would be required to identify these impurities related to the fabrication process, which would shed insights on the differences between simulated and the experimental peaks. However, since the main focus of this work is to demonstrate the LSPR characteristics of AuNI on different supporting substrates, the structural analysis of different ceramic substrates is not carried out here.

### 3.4. RI Sensitivity

**Figure 5:**
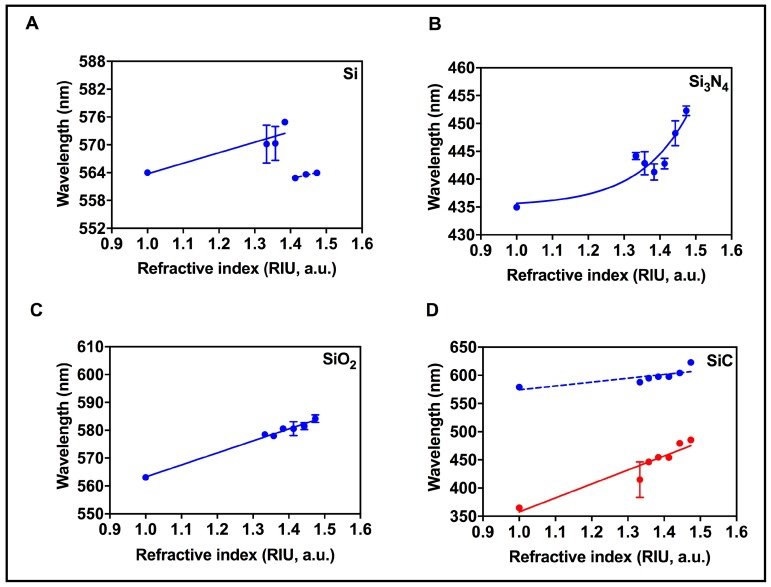
Refractive index characterization: The panels (**A**–**D**) display the changes in the LSPR wavelength of the AuNI when the refractive index varies from 1.00 to 1.47 on Si, Si_3_N_4_, SiO_2_ and SiC substrates respectively.

The developed AuNI on 4 supporting ceramic substrates were tested for refractive index sensitivity by exposing the top surface to air (RI=1.000), water (RI=1.333) and aqueous solutions of glycerol, with concentrations ranging 20–100 wt% (RI = 1.357–1.473). Red shifts in the characteristic LSPR peak are observed upon increases in the refractive index from 1.0 to 1.47 (Figure S2 in Supplementary Materials). Figure 5 displays the wavelength versus the refractive index of the 4 AuNI covered substrates. The curve fitting parameters for the sensitivities of AuNI are listed in Table 3. The wavelength increases with the increasing refractive index, leading to a red shift in the resonance characteristics for all 4 substrates, as shown in Figure 5A–D. Red shifts are primarily attributed to the increase in the local dielectric constant of the AuNI on all substrates. However, the trend of the red shifts in the resonance characteristics of Si, SiO_2_, Si_3_N_4_ and SiC is different from each other. Figure 5A shows that while the AuNI on the Si substrate produce a linear red shift upon increase in the refractive index, there is a discontinuous drop after RI=1.4. The interaction of the glycerol (<60 wt%) with AuNI on Si essentially removes some Au atoms from surface of the Si substrate. This causes a decrease in the size or/and an increase in the spacing of the AuNI when exposed to highly viscous glycerol solutions above 60 wt%, further leading to a change in the baseline of LSPR wavelength signal of AuNI during the RI measurements. On Si_3_N_4_ substrates (see Figure 5B), there is an exponential increase in the wavelength upon increase in the refractive index. It is also possible to fit the refractive index values with a linear line but only between RI = 1.333–1473. However, the linear fit in this range has a low regression coefficient of 0.71, suggesting that the developed nanoislands on Si_3_N_4_ present poor characteristics for RI sensing. Unlike the Si and Si_3_N_4_, the AuNI developed on the SiO_2_ substrate were found to be stable in viscous media (more than 60% concentration of glycerol) with a sensitivity of 43 nm/RIU and a good regression coefficient of 0.99 for refractive index sensing, see Figure 5C.

Figure 5D shows refractive index sensing on the SiC substrates. The two peaks of SiC substrate in air, one averaged at 364.85 nm (blue line) and the other at 579.06 nm (red line), are tested for refractive index sensitivity. It should be noted that we have considered the trialling edge of the peak shown in Figure 4 during simulations to compare the experimental refractive index , see details of experimental LSPR measurements in Figure S2 of supplementary data file. Both peaks are found to respond linearly to the change in the refractive index but with different sensitivities. In particular, the peak at 364.85 nm (red line) revealed a sensitivity of 247.80 nm/RIU in comparison with a sensitivity of 68.47 nm/RIU at 579.06 nm (blue line). Overall, from Figure 5 we can conclude that the RI-sensing response is different for all substrates even though the fabrication process of NI is the same. For instance the RI sensing characteristics on Si was found to be poor as compared to SiC. These reported characteristics of RI sensing, including enhanced sensitivity, stronger adhesion on a given substrate can be improved by changing the nanoisland fabrication parameters such as the thickness of the Au film and annealing time/temperature of the dewetting process. However, it should be noted that in this study we focus on revealing the effect of the supporting substrate of plasmonic nanostructures and therefore all parameters of the fabrication process except the supporting substrate are fixed in the experimental design.

### 3.5. RI Sensitivity and Morphology

**Figure 6:**
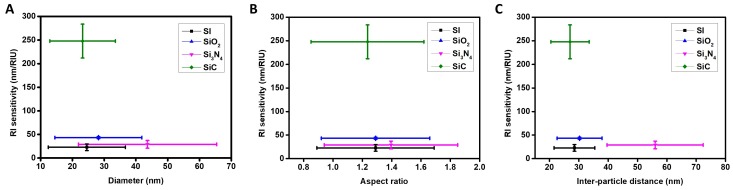
LSPR refractive index sensitivity of AuNI (1.00–1.47) plotted against the morphological variations of AuNI. (**A**) The Refractive Index (RI) sensitivity with respect to the mean diameter of AuNI. (**B**) Effect of the shape of the AuNI on LSPR sensitivity. (**C**) Effect of the inter-particle distance of NI.

Since the LSPR resonant frequency is dependent on the distribution and morphology of the plasmonic nanostructures, it is crucial to reveal the relationship between the RI sensitivity and morphological features of AuNI on different supporting substrates. Figure 6A–C exhibit the relation between RI sensitivity and the AuNI diameter, aspect ratio and inter-particle distance, respectively. The aspect ratio of the AuNI is the ratio of the major to minor axis of the AuNI (i.e., a perfect circle has the aspect ratio of 1.0). These morphological features of the AuNI were extracted directly from the SEM data shown in Figure 2. More specifically, the average and error values in Figure 6A–C are being reported from Figure 2E–G. The peak of the distribution function represents the average value and its full width at half maximum (FWHM) represents the error values. The linear fit for sensitivities of the AuNI in Figure 5 (Si: 22.74 ± 7.05 nm/RIU, Si_3_N_4_: 28.97 ± 8.18 nm/RIU, SiO_2_: 43.17 ± 2.07 nm/RIU and SiC: 247.80 ± 35.83 nm/RIU), are considered for the plots in Figure 6. Figure 6A indicates that there is no correlation between the sensitivity and the diameter of the AuNI. However, for substrates of Si, Si_3_N_4_ and SiO_2_, the larger variation in the diameter leads to larger deviation in the RI sensitivity. Similarly, from Figure 6B,C, small variations in the aspect ratio of the AuNI (more rounded shape) and the inter-particle distance yield the highest sensitivity among the Si, Si_3_N_4_ and SiO_2_ substrates. In contrast, the case for SiC cannot be compared with the other 3 substrates due to the anomalous emergence of multi-mode plasmonic resonant peaks as a result of the interaction between the AuNI and the supporting substrate of SiC. A possible explanation for this anomaly can be ascribed to the bulk defects in SiC which lead to optical non-linearity in the materials [46]. This non-linearity also results in high quality factor of the SiC substrate, further giving rise to the highest absorption of light energy when compared to the other three Si-based supporting substrates [47]. The non-linearity in SiC also induces 2 modes of absorption (more details in Section 3.3), one associated with LSPR at 452.7 nm and the other at 559.3 nm due to guided mode resonance evidenced from both experimental and simulation results in Figure 4. A detailed analysis of the morphological and optical characteristics of the SiC substrate would reveal further insights into this anomaly, which will be our future investigations.

## 4. Conclusions

We reveal that the refractive index sensitivity of plasmonic nanostructures depends on the type of the supporting substrate on which it is fabricated. Different dewetting process during the fabrication leads to variations in the shape, size and inter-particle distance among the plasmonic nanostructures. Even though the literature reports that the Au nanostructures of spherical shapes have a typical refractive index sensitivity of less than 100 nm/RIU, our results demonstrate that by simply changing the supporting substrate the sensitivity of the Au near spherical nanostructures can be enhanced, up to 247.80 nm/RIU when SiC is used as a supporting substrate. These results are important for applications in biosensor design, where often the shape of the nanostructure cannot be comprised over low sensitivity of the nanostructures, either due to the fabrication constraints or its large surface area available for sensing. To reveal the underlying mechanism behind the variation in the RI sensitivity with different Si-based ceramic substrates, independent morphological and optical characterizations of each substrate should be performed in the near future. Our work may serve as a benchmark to develop plasmonic nanomaterials with high sensitivity by choosing the right supporting substrate for a wide range of bio/chemical sensing applications.

## Figures and Tables

**Table 1:** Curve fitting parameters for lognormal distributions of AuNI diameters (*D*), aspect ratios (AR) and inter-particle distances (IPD) on various silicon substrates.

		Si	SiO_2_	Si_3_N_4_	SiC
μ	*D*	3.27	3.41	3.85	3.20
	AR	0.28	0.28	0.36	0.24
	IPD	3.35	3.42	4.05	3.31
σ	D	0.46	0.45	0.46	0.41
	AR	0.27	0.25	0.29	0.28
	IPD	0.22	0.22	0.26	0.21

**Table 2:** Contact angle measurements on ceramic substrates depending on AuNI deposition.

Substrate	Without AuNI (${}^{\circ }$)	With AuNI (${}^{\circ }$)
SiC	98.93–100.61	110.58–113
Si_3_N_4_	45.27–48.81	102.00–103.08
Si	73.89–74.37	110.95–109.38
SiO_2_	73.49–74.10	95.93–99.55

**Table 3:** Curve fitting parameters of the sensitivities of AuNI for refractive index characterization.

	RI Range	Type	Equation	Slope
Si	1–1.4	Linear, R sq. = 0.84	λ = 22.74RI + 541	22.74 ± 7.05 nm/RIU
	1.4–1.473	Linear, R sq. = 0.94	λ = 18.09RI + 537.04	18.98 ± 4.55 nm/RIU
Si_3_N_4_	1–1.473	Exponential	-	-
SiO_2_	1–1.473	Linear, R sq. = 0.99	λ = 43.71RI + 520.1	43.17 ± 2.07 nm/RIU
SiC	1–1.473	Linear, R sq. = 0.91	λ = 247.80RI + 109.9	247.80 ± 35.83 nm/RIU
	1–1.473	Linear, R sq. = 0.63	λ = 68.47RI + 35.83	68.47 ± 23.36 nm/RIU

## Supplementary Materials

The following are available online at https://www.mdpi.com/2079-4991/9/11/1530/s1, Figure S1: Contact angle measurements on SiC, Si_3_N_4_, Si and SiO_2_ substrates before and after the deposition of AuNI, Figure S2: LSPR wavelength peaks vary with different refractive index fluids, for 4 different silicon-based supporting substrates, Table S1: Contact angle measurement on ceramic substrates (Error represents standard deviation, N = 3).
