# Supplementary material for: Dewetting Metal Nanofilms—Effect of Substrate on Refractive Index Sensitivity of Nanoplasmonic Gold
Source: Nanomaterials (Basel). 2019 Oct 27;9(11):1530. doi: 10.3390/nano9111530 (PMC6915419; doi:10.3390/nano9111530)
Supplement: Supplementary file 1 [file nanomaterials-09-01530-s001.pdf]

Electronic Supplementary Material (ESI)

Dewetting metal nanofilms: Effect of substrate on refractive index sensitivity of nanoplasmonic gold

*Nikhil Bhalla[a,d], Aditya Jain[b], Yoonjoo Lee[c], Amy Q. Shen[*d] and Doojin Lee[*e,f]*

[a]*Nanotechnology and Integrated Bioengineering Centre (NIBEC), School of Engineering, Ulster University, Jordanstown, Shore Road, BT37 0QB, Northern Ireland, United Kingdom.*

[b]*Seagate Technology LLC, 7801 Computer Ave, Bloomington, MN 55435, USA.*

[c]*Energy Efficiency Materials Center, Korea Institute of Ceramic Engineering and Technology, 101 Soho-ro, Jinju-si, Gyeongsangnam-do, 52851, South Korea.*

[d]*Micro/Bio/Nanofluidics Unit, Okinawa Institute of Science and Technology, Onna, Okinawa 904-0495, Japan.*

[e]*Department of Polymer Science and Engineering, Chonnam National University, Gwangju, 61186, South Korea.*

[f]*Fibrous Ceramics and Aerospace Materials Center, Korea Institute of Ceramic Engineering and Technology, 101 Soho-ro, Jinju-si, Gyeongsangnam-do, 52851, South Korea.*

∗Email:dlee@chonnam.ac.kr, amy.shen@oist.jp

1. Contact angle measurements

Contact angle measurements were performed using the contact angle instrument (Theta Attension, Biolin Scientific, USA). A drop of 10 µl was dispensed on the ceramic substrate and the resting contact angle was measured by the in-built software of the instrument. The contact angle on the ceramic substrates, measured before and after the deposition of the gold nanoislands (AuNI) is summarized in figure S1 and table 1.

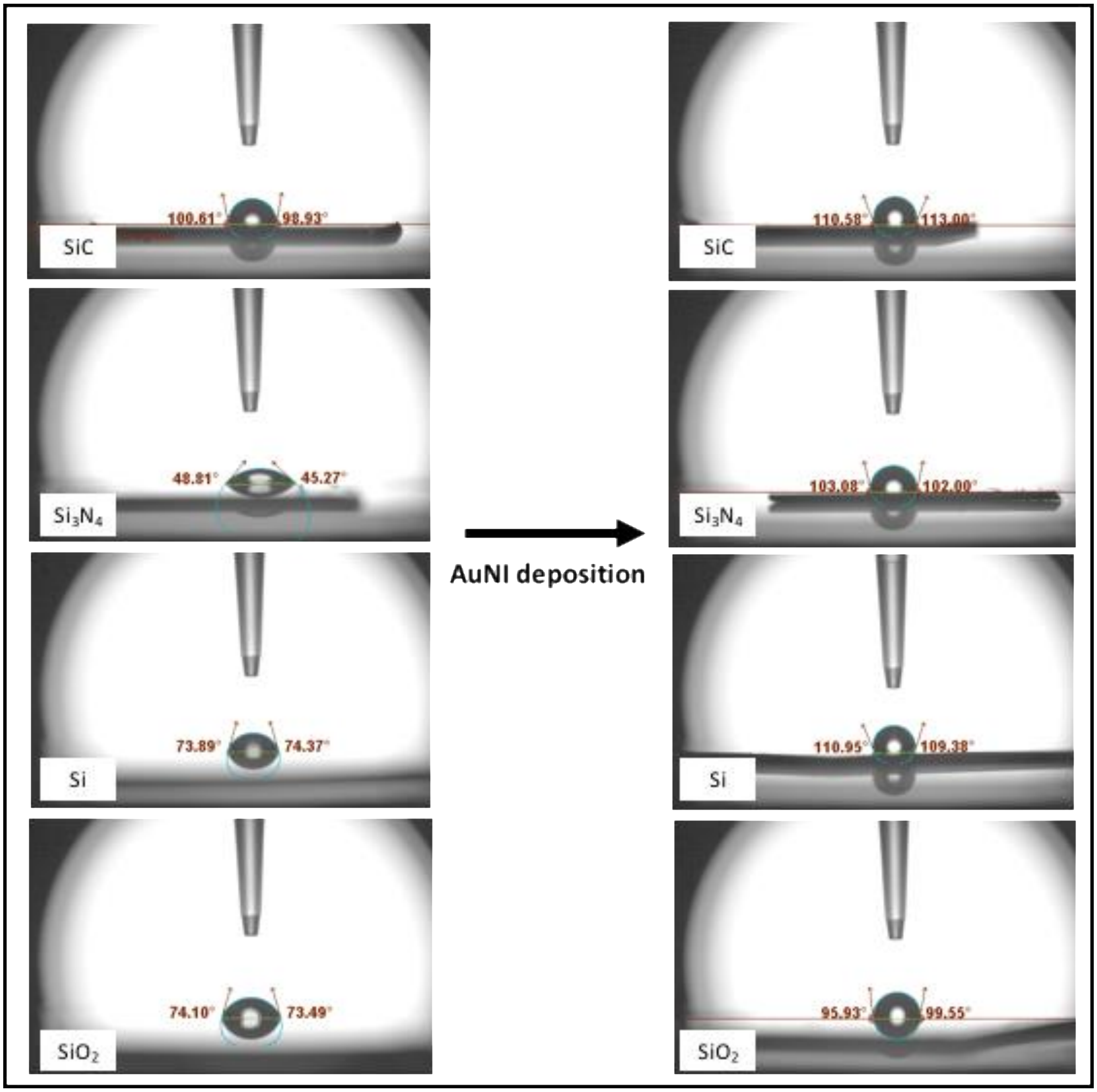

Figure S1. Contact angle measurements on SiC, Si_3N_4, Si and SiO_2 substrates before and after the deposition of AuNI.

Table S1. Contact angle measurement on ceramic substrates (Error represents standard deviation, N = 3)

Material	Without AuNI (°)	With AuNI (°)
SiC	99.77 ± 0.84	111.79 ± 1.21
Si3N4	47.04 ± 1.77	103.04 ± 0.04
Si	74.13 ± 0.24	110.16 ± 0.78
SiO2	73.79 ± 0.30	97.74 ± 1.81

2. Refractive index sensitivity

Figure S2 shows the changes in the wavelength of the LSPR peak of AuNI fabricated on Si, SiO_2, Si_3N_4 and SiC substrates upon exposure to solutions (water and glycerol 20-100 wt%) of varied refractive indices. Red shifts in the characteristic LSPR peak are observed upon increase in the refractive index from 1.0 to 1.47. The red shifts observed are plotted in figure 5 and discussed in detail in the section 3.4 of the main manuscript.

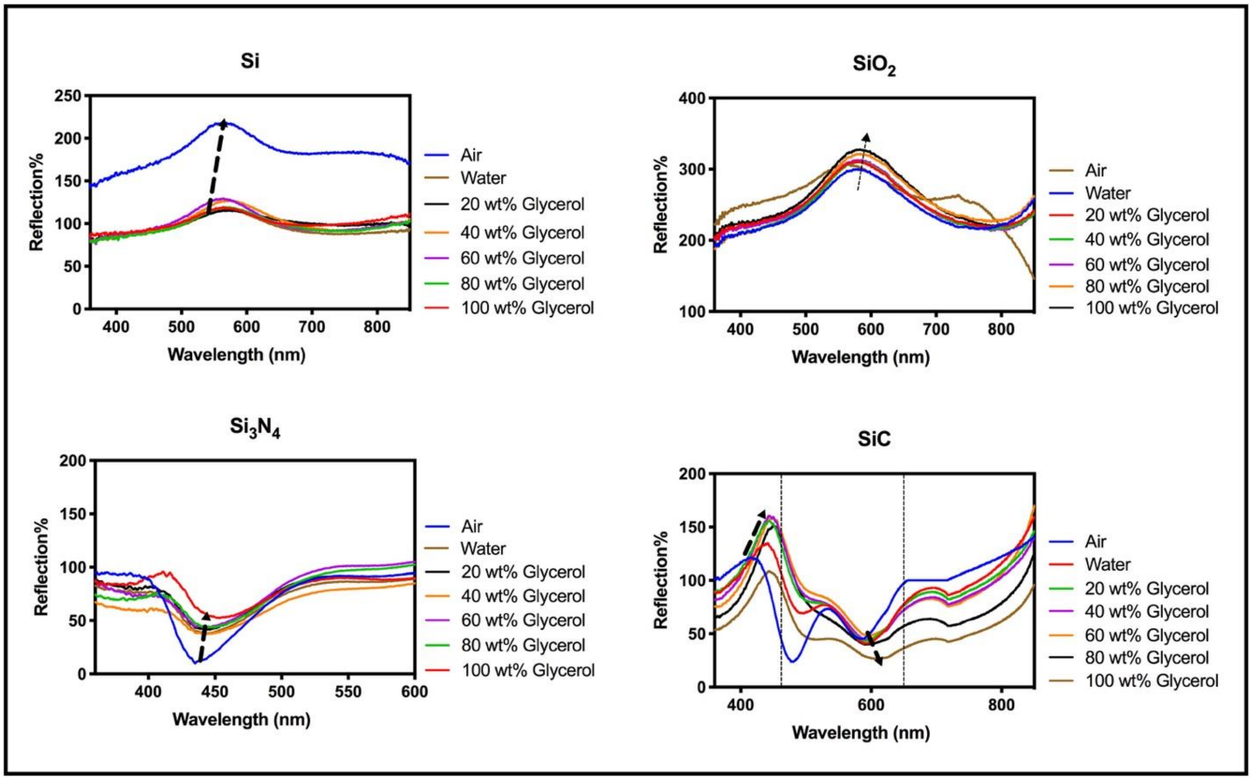

Figure S2: LSPR wavelength peaks vary with different refractive index fluids, for 4 different silicon-based supporting substrates.
